# The *Sorbus* spp.—Underutilised Plants for Foods and Nutraceuticals: Review on Polyphenolic Phytochemicals and Antioxidant Potential

**DOI:** 10.3390/antiox9090813

**Published:** 2020-09-01

**Authors:** Viive Sarv, Petras Rimantas Venskutonis, Rajeev Bhat

**Affiliations:** 1 ERA Chair for Food (By-) Products Valorisation Technologies of Estonian University of Life Sciences -VALORTECH, Estonian University of Life Sciences, Fr. R. Kreutzwaldi 1a, 51014 Tartu, Estonia; rimas.venskutonis@ktu.lt (P.R.V.); rajeev.bhat@emu.ee (R.B.); 2 Institute of Agricultural and Environmental Sciences, Polli Horticultural Research Centre, 69108 Polli, Estonia; 3 Department of Food Science and Technology, Kaunas University of Technology, Radvilėnų pl. 19, LT-50254 Kaunas, Lithuania

**Keywords:** rowan, phytochemical composition, bioactivities, health benefits, food applications

## Abstract

The *Sorbus* spp. are valuable plants, which have been used for ornamental purposes, in traditional medicines and less seldom in foods. Recent studies have revealed different anatomical parts of the *Sorbus* spp. to contain valuable phytochemicals demonstrating various bioactivities. However, in terms of applications in the products intended for human consumption, *Sorbus* still remains as an underutilised genus. The increasing number of studies on phytochemicals, antioxidant potential and other bioactivities of *Sorbus* extracts has revealed the prospects of expanding its use in natural medicines, cosmetics and as innovative food ingredients, which might find wider applications in functional foods and/or nutraceuticals. Caffeoylquinic acids, flavonoids and proanthocyanidins have been reported in various *Sorbus* spp. as the most abundant polyphenolic antioxidants. The preparations of various plant anatomical parts have been used in ethnopharmacology as natural remedy for treating bacterial, viral, inflammatory diseases including tumors. *Sorbus* spp. plant parts have also been tested for management of diabetes, neurological, and cardiovascular disorders. The present review is focused on *Sorbus* plants (in total 27 *Sorbus* spp.), their composition and properties in terms of developing promising ingredients for foods, nutraceutical, cosmeceutical and other applications. It is expected that this review will assist in designing further studies of rowans and other *Sorbus* spp. in order to expand their uses for various human applications.

## 1. Introduction

During the past few decades, search and development for novel highly valued bioactive compounds from plants has become a topical issue for researchers, health professionals, producers, and consumers. Considering vast number of species in the Plant Kingdom, there are still infinite number of under explored plants, which may serve as an excellent platform for discovery of new compounds and developing valuable preparations. Underutilised plants have become of a particular interest in the era of functional foods, nutraceuticals and personalized nutrition. Thus, natural bioactive compounds can play the most important role in the development of health promoting products based on individual genome and/or microbiome [1,2].

Fruits and vegetables have been considered as healthy foods, mainly owed to the presence of high amounts of valuable nutrients such as vitamins, minerals, polyphenolic antioxidants, dietary fibre and others. In this regard, many well-known comprehensively valorised and globally commercialized berry fruits such as raspberries, strawberries, black currants, blueberries, cherries and others are among the richest sources of vitamins and bioactive phytochemicals, particularly antioxidant polyphenols. The above-mentioned berries also possess characteristic and highly appreciated sensory properties and hence are consumed both as fresh fruits and/or in processed forms. However, there are still many underutilised berries, mainly due to their specific and therefore non-acceptable for consumers flavour.

The *Sorbus* spp. (common names rowans, whitebeams and others) are deciduous shrubs or trees, which although being widely grown in the gardens and parks, can be assigned to the underutilized plants in terms of their applications as foods, nutraceuticals and/or cosmeceuticals. The rowans are the most widely studied *Sorbus* spp. Wild rowan trees are tolerant to harsh Nordic climate and poor growing environment such as rocky and windy slopes and even the mountains and may reach up to 15 m height.

Other anatomical parts of berry producing plants may also contain valuable phytochemicals; therefore, bark, leaves, inflorescences have been empirically used in folk medicines for centuries. The bark of the *Sorbus* trees is mostly smooth, lustrous, dark, with elongated horizontal lenticels; the leaves are pinnately compound, the leaflets toothed or rarely entire, while the inflorescences may be extra-large, convex panicles [3]. The interest in *Sorbus* spp. as a promising source of valuable phytochemicals has increased during last decade. For instance, in the Clavirate Analytics Web of Science database, out of 133 publications with the search words ‘*Sorbus* + antioxidants’ 105 have been included since 2010; while in the same period 68 records out of 91 have been found with the search words ‘*Sorbus* + polyphenolics’ (accessed on 20 July 2020). Comprehensive review on *Sorbus* phytochemicals has been recently published; it focuses on *Sorbus* as an ethnopharmacologically important but underestimated genus and provides extensive information on plant phytochemicals [4]. The present review focuses on *Sorbus* composition and properties in terms of development of promising ingredients for food, nutraceutical, cosmeceutical and other applications. For this purpose, it includes some important information; for instance, more detailed data on until now reported concentrations of different polyphenolic phytochemicals and the values of antioxidant potential in different *Sorbus* spp. This information might assist in selecting the most promising species/cultivars and their anatomical parts for further studies and applications.

## 2. Botanical Classification and General Uses

**Figure 1:**
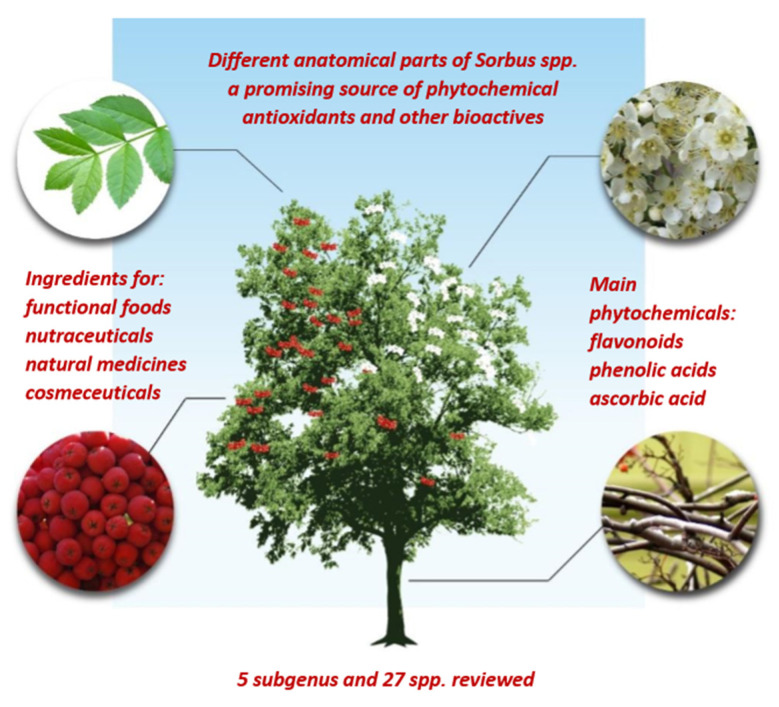
Potential uses of different parts of *Sorbus* spp.

The copious genus *Sorbus* L. (Rosaceae, Maloideae) covers up to 250 species, which in addition are divided into 6 subgenus, namely *Sorbus*, *Aria*, *Micromeles*, *Cormus*, *Tominaria*, and *Chamaemespilus*. According to Robertson et al. [3] approximately 35 species exist in the Caucasus and Turkey, 91 in Europe, and 111 in China, Vietnam, Myanmar, and in the Himalayas. The bitter fruits of wild rowan are round in shape and they can be red, orange, yellow, pink or white with homogeneous flesh (Figure 1) [3]. The rowan tree can yield up to 20 kg of rowanberries [5]. Traditionally, people consumed rowanberries in small amounts as a mash to improve the appetite and stimulate production of gastric acid. In folk medicine these fruits have been used as a laxative, against rheumatism and kidney diseases, and gargle juice against hoarseness [6]. Rowan berries have been traditional diuretic, vasodilatory, anti-inflammatory, anti-diarrheal remedies and a source of ascorbic acid (vitamin C); in some countries they also have been used for treating intestinal obstructions, various liver and gallbladder diseases [7]. The leaves have sometimes been used to feed livestock while the fruits have been administered to domestic pigs and goats against bacterial infections [8]. In order to make the selection of abundant genus *Sorbus* the species listed in the United States Department of Agriculture (USDA) database [9] were used in the current review. In addition, the species with a more comprehensively investigated bioactivity were included.

The subgenus *Sorbus*, commonly noted as a mountain ash (Amur or European mountain ash), rowan or quick beam, is distributed in the Northern Hemisphere. It has hairless or thinly hairy leaves [3]. This review covers 19 species from the large *Sorbus* subgenus (Table 1): *S. americana* Marshall (American mountain ash), *S. aucuparia, S. californica* Greene (California mountain ash), *S. cashmiriana* Hedl., *S. commixta* Hedl. (The Japanese rowan)*, S. decora* C.K. Schneid, (the northern mountain ash)*, S. dumosa* Greene (Arizona Mountain Ash), *S. gracilis* (Sieb. & Zucc.) K. Koch., *S. groenlandica* (C.K. Schneid.) A. Löve & D. Löve (the Greenland mountain-ash), *S. koehneana* C.K. Schneid. (Koehne mountain ash), *S. pohuashanensis* (Hance) Hedl., *S. pogonopetala* Koehne, *S. sambucifolia* (Cham. & Schlecht.) Roem. (Siberian Mountain-ash), *S. scalaris* Koehne, *S. scopulina* Greene, *S. setschwanensis* (C.K. Schneid.) Koehne, *S. sitchensis* M. Roem (western mountain ash), *S. tianschanica* Rupr., *S. wilfordii* Koehne. Different anatomical parts of these species have been used for medicinal and food purposes (Figure 1). The leaves of *S. tianschanica* have been used to treat asthma, ventricular myocytes, dyspnoea, tuberculosis and gastritis [10], while both the leaves and the bark of *S. decora* are known as an antidiabetic medicine [11]. The bark of *S*. *americana,* due to hypo-glycaemic properties has also been used for treating diabetes; while other applications include vaso-relaxant, antitussive and tonic activities [12]. In oriental medicine, the stems and bark of *S*. *commixta* have been used to treat arthritis and inflammatory diseases and as hypoglycaemic, vasorelaxant, antitussive and tonic agents [13,14]. The bark preparation of *S*. *cashmiriana* has been used to treat nausea and heart diseases, while its berries have been used to cure scurvy [15]. The fruits, stems and bark *S*. *pohuashanensis* have been widely used in traditional Chinese medicine for treating chronic tracheitis, tuberculosis and oedema [16]. The fruits of *S*. *sambucifolia* have been used in drinks and foods (beverages, jams, jellies, floured dried fruit, etc.), while for medicinal purposes—in case of avitaminosis, arteriosclerosis and as antipyretic or diuretic agent. Indigenous people used to eat fresh *S. scopulina* berries; however, currently they are sometimes used in pies, preserves, or wine-making [17]. In folk medicine, the fruits and the inflorescences of *S. aucuparia* (European rowan) have been used as traditional anti-inflammatory, antidiarrheal, vasodilatory and an appetite-improving agents, as well as a good source of vitamins, diuretic and mild laxative medicine [18,19]. In traditional Austrian medicine, the tea, syrup, jelly or alcoholic tincture of *S. aucuparia* fruits have been used to treat fever, infections, colds, flu, rheumatism and gout [20].

From the subgenus *Aria* with 39 species, commonly known as whitebeams, 6 species, namely *S. aria* Crantz, *S. intermedia* (Ehrh.) Pers., *S. norvegica* Hedl., *S. folgneri* (Schneid.) Rehd., *S latifolia* (Lam.) Pers, *and S. minima* (Ley) Hedl. are covered in this review. These species have simple white-hairy leaves and are distributed in the temperate regions of Europe and in Asia. Traditionally, the leaves of *S. aria* were consumed as antidiarrheal ingredients, while their berries have been used in jellies, jams, brandy, liqueurs, conserves and vinegar, as traditional bread flour extender, diuretic, anti-inflammatory, anti-diarrhoeal, vasodilatory agent and vitamin source [21]. Moreover, the fruits and inflorescences of *S. aria* have been used as a diuretic, laxative and emmenagogue folk medicine for treating painful menstruation, constipation and kidney disorders [22]. The berries of *S. intermedia* have been added to bread in Estonia [23], while the berries of *S. norvegica, S. folgneri, S latifolia* and *S. minima* were tested for their α-amylase and α-glucosidase inhibitory activities [24].

The subgenus *Micromeles*, commonly known as Korean whitebeam, alder-leafed whitebeam, contains around 25 narrow leaved species of shrubs and trees with white flowers, distributed from Nepal to the South Kuriles, extending to the Malay Peninsula and Sumatra [25]. In the Korean folk medicine the twigs of the most widely distributed species, *S. alnifolia* (Siebold & Zucc.) K. Koch., were used for treating neurological disorders [26].

*Aria*, *Micromeles* and *Chamaemespilus* have simple leaves and pomes with groups of tanniferous cells, however, *Chamaemespilus* (false medlar or dwarf whitebeam) differs by a rather different flower shape [27]. *Chamaemespilus* is not reviewed in the current study due to the lack of information about its uses and antioxidant activity.

The subgenus *Torminaria* (common names wild service tree, chequers, and checker tree) with three species is distributed in the temperate Europe, south to the mountains of North Africa and east to the Caucasus ranges. It has maple-like simple, 3-5-lobed leaves and brown pomes without groups of tanniferous cells. The fruits of *S. torminalis* have been traditionally used as diuretic or anti-inflammatory, antidiarrheal (dried), vasodilatory remedy and as a source of vitamins [27,28]. In the current work, the uses and antioxidant potential of 2 varieties of *S. torminalis*, (var. *torminalis* and *semitorminalis*) are surveyed.

The subgenus *Cormus* with pomes without starch and groups of tanniferous cells [27] and compound leaves, is distributed in the warm-temperate Europe, North Africa and Asia. Unlike the subgenus *Sorbus*, *Aria* and *Torminaria*, whose fruit carpels are not fused, subgenus *Cormus* is with distinct fused carpels in the fruit. In this review only *S. domestica*, also known as true service tree or sorb tree [29], is included. It has been reported that the fruits of *S. domestica* are traditional anti-inflammatory, antidiarrheal (dried), antidiabetic, diuretic, vasodilatory agents and vitamin source [30].

Although, wild rowanberries are sour in taste they still contain a wide array of healthy components. In the beginning of the 20th century, the Russian practitioner Michurin started the breeding program with *S. aucuparia* to improve the flavour and increase the fruit mass of rowanberries. Crossbreeding of rowan with the *Malus*, *Mespilus*, *Aronia*, or *Pyrus* spp. produced interesting sweet-fruited rowan hybrids. These new hybrids have been bred particularly for northern conditions and they have demonstrated great frost-resistance in the Nordic countries [31]. The famous crossbreeds of *S. aucuparia* in Russia were called ‘Burka’, ‘Likjornaja’, ‘Dessertnaja’, ‘Granatnaja’, ‘Rubinovaja’, and ‘Titan’ [32]. The Western European hybrids of *S. aucuparia* include ‘Apricot Queen’, ‘Brilliant Yellow’, ‘Chamois Glow’, ‘Pink Queen’, and ‘Salmon Queen’ [33]. In contrast to wild rowanberries, the hybrids are much more palatable [34] and the sugar content in their cultivars is 1.2–2.1 times higher than in the wild rowanberries [35]. In the current review, the bioactivity and phytochemical contents of several *S. aucuparia* cultivars are compared with the wild berries.

## 3. Nutritional Composition

Wild rowanberries are not consumed as fresh fruits due to their specific astringent taste, imparted mainly by the tannins. These cause the dry feeling in the mouth when consumed. Therefore, they have rather limited applications for producing food products. However, due to the nutritive value and health benefits the berries of *S. aria*, *S. aucuparia*, *S. domestica*, *S. sambucifolia*, *S. scopulina*, and *S. torminalis* have been traditionally used for pressing juice, in alcoholic beverages, purees, jams and jellies [28,35]. These benefits are due to the significant amounts of phytochemicals, such as vitamins, carotenoids, and phenolic acids as well as important in nutrition minerals, iron, potassium, and magnesium. In addition, rowanberries contain a sweet-tasting sugar alcohol sorbitol, which slowly metabolizes in the human body and therefore is suitable as a sweetener for people suffering from diabetes [45].

It was reported that rowanberries contain 3-fold higher amount of ascorbic acid than oranges [5]. For instance, Mrkonjić et al. [28] determined approximately 0.1 mg/g d.w. (dry weight) of ascorbic acid in *S*. *aucuparia* berries and 0.42 mg/g dw in fruit jam. The recommended dietary allowance of ascorbic acid is 60 mg per day, while 5–7 mg a day prevents scurvy. Tocopherols are important fat-soluble vitamins in rowanberries. The mean concentrations of vitamin E activity demonstrating α-tocopherol, δ-tocopherol, and γ-tocopherol in *S*. *aria* and *S. aucuparia* were reported 2.82, 0.11, 2.01 µg/g dw and 4.89, 0.58, 1.71 µg/g dw, respectively [46]. Klavins et al. [47] determined even higher content of α–tocopherol (3.34 µg/g dw) in *S. aucuparia* fruit, while the content of γ-tocopherol was remarkably lower, 0.25 µg/g dw. The recommended intake of vitamin E for adults is in the range of 7 to 15 mg per day. The epidemiological studies showed that humans who consumed vitamin E richer foods had lower incidence of cancer, dementia and/or cardiovascular diseases [48].

In nature, β-carotene, a precursor (inactive form) of vitamin A, is a strongly coloured red-orange pigment, which is abundant in some plants and fruits. Berņa and Kampuse reported that *S. aucuparia* contains 2.5 mg of total carotenoids per 100 g [49]. The average daily intake of the strong antioxidant β-carotene is in the range of 2–7 mg, as estimated from a pooled analysis of 500,000 women living in the US, Canada, and some European countries [50]

The minerals are important for all living organisms. Aslantas et al. reported high content of 8 essential minerals in *S. aucuparia* (in mg/100 g): potassium, 154; phosphorus, 12.3; calcium, 29.9; magnesium, 27.84; iron, 2.42; copper, 0.294; zinc, 0.861; and manganese 0.503 [51]. The tree bark of *S. domestica* has been reported as a good source of Ca, Zn, Fe, while the seeds were rich in K, Mg, Fe and Zn [30]. Plant oils are important as food ingredients and as a source of essential fatty acids for human nutrition. In seed oils of *S*. *aucuparia* the sum of linoleic and oleic acids exceeded 90% of the total fatty acids [37]. Ivakhnov et al. [52] optimized the procedure for oil extraction from *S. aucuparia* alcoholic beverage production waste using the supercritical CO_2_ as a solvent and recovered 9.02% (w/w) high quality oil.

## 4. Total Phenolic Content and Quantitative Composition of Phytochemical Antioxidants in *Sorbus* spp.

### 4.1. Total Phenolic Content

In general, the leaves and inflorescences of *Sorbus* spp. were reported to contain higher amounts of the total phenolic content (TPC) than the fruits (Table 2). Usually TPC is expressed in gallic acid equivalents (GAE). Thus, the highest TPC was reported in the dried leaves of *S. wilfordii* (12.31% GAE), as well as in the inflorescences of *S. aucuparia* (11.83% GAE) [53]. Predominantly, in the tested plant parts of the *Sorbus* spp., the total level of phenolics was significantly higher in the inflorescences than in the leaves [54], except for *S. gracilis*, when the TPC in the leaves was slightly higher than in the inflorescences, 11.06 and 10.72% GAE, respectively [53]. The highest TPC in fruit was detected in *S. aria* (2.98% GAE) dw; the fruits of *S. aucuparia* and *S. intermedia* contained only slightly lower TPC, 2.68% and 2.24% GAE dw, respectively [54]. The lowest TPC values among the tested *Sorbus* spp. was found in the *S. americana* fruits; it was only 3.60–5.39 mg/g dw [54,55]. Gaivelyte et al. analysed leaf and fruit material of 10 *Sorbus* spp. and 9 cultivars and found that the TPC varied approximately 5 times, both in leaf and fruit samples, i.e., in the range of 7.18–35.74 mg/g and 2.24–11.19 mg/g, respectively [56]. The berries of *S. aria* and *S. aucuparia* grown at different altitudes were compared; however, there was no correlation between TPC, total proanthocyanidins, radical scavenging capacity and growing site. Nevertheless, slightly higher TPC values were observed in *S. aucuparia*, while *S. aria* had higher content of proanthocyanidins [57].

TPC may highly depend on berry maturity, while the recovery of phenolics depends on extraction solvent. For instance, the diethyl ether fraction separated from the crude methanol extract isolated from fruit pulp of *S. domestica* berries matured at room temperature for 1 week had the highest TPC [58]. Bobinaitė et al. [59] reported the TPC in acetone, ethanol and water extracts of rowanberry pomace, which was almost similar, 10.94, 10.43 and 9.60 mg/g, respectively; while the content of individual compounds depended remarkably on the applied solvent. It was suggested that considering only slight differences in the recovery of total phenolics between the applied solvents, water would be the most attractive due to the price, availability and safety.

Olszewska et al. [37] investigated the effects of extraction with chloroform and 70% methanol and fractionation with diethyl ether, ethyl acetate, *n*-butanol of soluble in different solvents substances present in inflorescences and leaves of 7 *Sorbus* spp., namely *S. aucuparia, S. commixta, S. decora, S. gracilis, S. koehneana, S. pogonopetala* and *S. wilfordii*. N-butanol and ethyl acetate were the most effective in recovering antioxidants from *Sorbus* leaves, whereas ethyl acetate, *n*-butanol and diethyl ether fractions of *S. pogonopetala* and *S. wilfordii* leaves contained the highest TPC, 39.56–58.17% dwe (dry weight of extract)**.**

### 4.2. Phenolic Acids

Chlorogenic (3-*O*-caffeoylquinic acid, 3-CQA) and neochlorogenic acids (5-*O*-caffeoylquinic acid, 5-CQA) are the main phenolic acids reported in *Sorbus* spp. [53,55,57]. Moreover, it has been reported that caffeoylquinic acids constitute 56–80% of the total phenolics in *Sorbus* fruits, whereas the cultivated berries contain less caffeoylquinic acids than wild rowanberries [60]. The content of chlorogenic acid in the berries of *S. aucuparia* was up to 10.01 mg/g dw [57], while the content of neochlorogenic acid in the tested 5 cultivars was up to 7.31 mg/g dw [60]. Generally, the content of caffeoylquinic acids in the inflorescences was reported to be higher than in the leaves or berries. The predominant caffeoylquinic acid in the all assayed inflorescence samples was chlorogenic acid [54,55,56,57,60] with the highest concentration in *S. sambucifolia,* 4.17% dw [53] the highest contents of neochlorogenic acid were in the inflorescences of *S. koehneana* (1.98%), *S. decora* (1.26%) [53], and *S. aucuparia* (1.37%) [54]. The concentrations of chlorogenic acid in water and methanol extracts, as well as in the jam of *S. aucuparia* were 5.69, 5.80 and 2.60 mg/g dw, respectively [28]. It seems that some species instead of chlorogenic acids biosynthesize ferulic acid as the major one; in the methanol and water extracts and jams of *S. torminalis* its content was up to 62.6 µg/g dw [28].

In addition, ferulic acid content was reported in the leaves of some *Sorbus* spp., such as *S. aucuparia*, *S. aria* [43] and *S. subfusca* [61]. The methanol and water extracts and jams of both *S. torminalis* var. *torminalis* and *semitorminalis* also contained up to 23.2 µg/g dw protocatechuic acid, while in the jam of *S. aucuparia* its concentration was 12.5 µg/g dw. Protocatechuic acid was also reported in the fruits, leaves and bark of *S. alnifolia* [62], in the extracts of *S. aucuparia*, *S. commixta, S. gracilis, S. decora* and *S. koehneana* inflorescences [37], in the extracts of *S. gracilis*, *S. pogonopetala, S. wilfordii* [37], *S. domestica* leaves [63] and in the *S. domestica* fruit pulp [41]. Gallic acid was found only in the water extract of *S. torminalis* var. *semitorminalis* in concentration of 5.69 µg/g dw [28].

Some other well-known phenolic acids and their derivatives such as cinnamic, vanillic, *p*-coumaric and benzoic acids have been found in traces in the fruits of *S. aucuparia* [64] and *S. domestica* [41], while *p*-coumaric acid was also detected in the *S. discolor* berries [40]. Caffeic acid and its derivatives were reported in the berries of *S. aucuparia* [40], *S. domestica* [62], *S. discolor* [40], *S. alnifolia* [62], *S. pohuashanensis* [16], *S. torminalis* [40]. Vanillic acid was found in the leaves of *S. aria* [40], coumaric acid in the inflorescences of *S. aucuparia, S. commixta, S. decora, S. gracilis, S. koehneana* and in the leaves of *S. domestica* [63], *S. pogonopetala, S. gracilis,* and *S. wilfordii* [37].

### 4.3. Flavonoids

**Figure 2:**
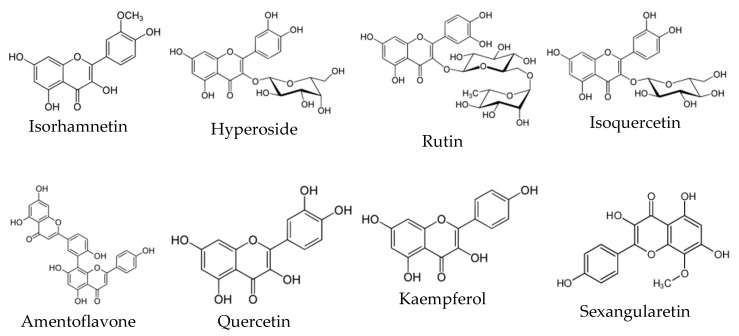
The structures of the main *Sorbus* flavonoids.

Quercetin, kaempferol, isoquercetin, rutin, hyperoside and isorhamnetin were reported in the samples of selected *Sorbus* fruits, leaves and inflorescences as the major flavonoids (Figure 2). Quercetin was the predominant flavonoid in all selected leaf and inflorescence samples and the highest values were found in the inflorescences of *S. aucuparia* (1.11% dw) followed by *S. intermedia* (1.05% dw) [18]. Among the leaf samples the highest content of quercetin was determined in *S. aucuparia* and *S. wilfordii*, 0.88% and 0.90% dw, respectively [53]. The content of quercetin in the fruits of *S. aucuparia, S. intermedia, S. aria* was 0.51, 0.31, 0.09 mg/g, respectively [54]. The highest content of isoquercetin was found in *S. commixta* fruits and leaves, 0.65 mg/g and 5.24 mg/g, respectively; among analysed rowanberries, the fruits of the same species had the highest content of hyperoside, 1.19 mg/g [56]. Kaempferol was quantified in the fruits, leaves and inflorescences of *S. aria, S. aucuparia* and *S. intermedia*; the highest content of this flavonoid was present in *S. aucuparia* [54]. The leaves of *S. setschwanensis* and *S. aria* were also rich in kaempferol, which constituted 0.31% [53] and 0.26% dw [54], respectively. Isorhamnetin was found only in the fruits [40], leaves and inflorescences of *S. torminalis* [28], *S. intermedia* and *S. aria* [54]. Some isorhamnetin conjugates were also identified in *S. discolor* [40] and *S. domestica* [18]. Olszewska et al. using bioactivity-guided assay isolated several flavonoids, such as isorhamnetin 3-*O*-β-glucopyranoside, astragalin, isoquercitrin, hyperoside, kaempferol 3-*O*-β-glucopyranoside-7-*O*-α-rhamnopyranoside, quercetin 3-*O*-β-glucopyranoside-7-*O*-α-rhamnopyranoside, rutin, from the leaves of *S. aria* [65]. Among 10 investigated fruit samples of *S. aria* and *S. aucuparia* the highest content of rutin was found in the *S. aria* fruits reaching up to 892 µg/g dw [57]. Rutin was also abundant in the leaves of *S. anglica* [56].

Sexangularetin was one of the most abundant flavonoid component in the inflorescences; *S. aucuparia* and *S. scalaris* contained 0.19% and 0.14% dw, respectively [53]. Epicatechin was reported in the leaves of many *Sorbus* spp. [71,72], as well as in the berries of *S. aucuparia* [60] and *S. torminalis* var. *semitorminalis* [28]. Hesperidin was found only in the leaves of *S. tianschanica* [67,73]. The highest levels of proanthocyanidins among the inflorescences of 12 tested species were found in the *S. pohuashanensis* and *S. sitchensis*, 7.67% and 7.14% CyE, respectively. Among the 17 leaf samples, *S. gracilis* had the highest concentration of proanthocyanidins, 6.56% CyE [53]. Among the rowanberries, the highest content of proanthocyanidins was found in the fruits of *S. aria,* 1.80% CyE [54]. Catechin and epicatechin were the main flavonoid components in the samples of *S. decora* stembark [74], rootsock [75], but also in the water extract of *S. torminalis* var. *semitorminalis* [28].

Quercetin content in methanol extract of *S. torminalis* var. *semitorminalis* was 11.0 µg/g while in water extract it was 2-fold lower, 6.53 µg/g [28]. The content of rutin in water and methanol extracts of *S. aucuparia* was found similar, 82.3 and 80.4 µg/g dw, respectively [28]. Hydroethanolic (70%) extract of dried *S. commixta* stems and cortex contained higher by 50.43% total polyphenol and flavonoid content than water extract; the former also demonstrated stronger antioxidant capacity [68]. Exceptionally high content of amentoflavone was found in *S. torminalis* var. *semitorminalis* water and methanol extracts as well as in its jam, namely 362, 974, and 195 µg/g dw, respectively. However, its content in the extracts and jam of *S. torminalis* var. *torminalis* and *S. aucuparia* differed just slightly: it was 15.8, 19.3 and 16.8 µg/g dw and 10.7, 11.9 and 8.4 µg/g dw, respectively [28]. Up to 119 µg/g dw of quercetin-3-*O*-glucoside were reported in *S. americana* [55]. Typically, anthocyanins have been detected in the *S. aucuparia* cultivars however only in low concentrations, usually less than 1% of the total phenolics in the wild fruits [60]. Bobinaitė et al. [59] reported that the total content of proanthocyanidins in *S. aucuparia* pomace water extract was 10.4 and 3.8 times, higher than that in the acetone and ethanol extracts, respectively.

## 5. Antioxidant Potential of *Sorbus* spp.

Plant material, suitable for cost-effective production of natural antioxidants should contain reasonable amount of polyphenolics (usually not less than 8–10% GAE/dw), demonstrate comparatively strong antioxidant properties in several assay systems and exhibit as low as possible toxicity, which should be acceptable for human applications [53].

Large number of phytochemicals belonging to various classes of organic compounds have been identified in various *Sorbus* spp. [4]. The presence of significant amounts of polyphenolic antioxidants, mainly flavonoids and phenolic acids, has also been reported in *Sorbus* spp. (Table 2). Moreover, many authors observed good positive correlation between the concentration of phenolics, e.g., the sum of proanthocyanidins, caffeoylquinic acids and flavonoid aglycones and antioxidant properties [31,57,58]. Therefore, in many studies rowanberries exhibited significant antioxidant activity (Table 3), which was comparable or in some cases even higher than that of many other edible berries, such as chokeberries and bilberries [76]. Various methods have been applied for assessing antioxidant properties of rowanberries and their extracts, most frequently using the in vitro radical scavenging capacity assays and inhibition of lipid peroxidation [77], reducing power, chain-breaking potential of radical reactions [60] and others. The majority of studies investigated *Sorbus* fruits, leaves and inflorescences; however, antioxidant properties of tree bark and seed oil were also reported [46].

The main polyphenolic compounds responsible for antioxidant properties of rowanberries are phenolic acids (mostly caffeoylquinic acids), flavonols (quercetin, isoquercetin, hyperoside, rutin, catechin, epicatechin), anthocyanins (mainly cyanidin or pelargonidin glycosides), and proanthocyanidins [53,60]. In addition, many studies have reported several quercetin, sexangularetin (SX) and kaempferol (KA) glycosides in the fruits, inflorescences, leaves and stems of various *Sorbus* spp. (Table 2).

The stage of maturity [45], genotype [40], species [53], geographic origin [44], climatic environment, as well as storage conditions [78] and treatment [28] affect the composition of bioactive constituents. For example, Mrkonjić et al. [28] reported that among 12 identified in *S. aucuparia* and *S. torminalis* phenolic compounds chlorogenic acid was the most abundant in the former, while flavonoid amentoflavone in the latter one. The fruits of *S. aucuparia* better scavenged DPPH^•^ (2,2-diphenyl-1-picrylhydrazyl), ^•^NO, O_2_^•^, HO^•^ and inhibited lipid peroxidation (LP) than those of *S. torminalis*; however, both varieties of the latter species, namely *torminalis* and *semitorminalis* demonstrated almost identical antioxidant potential.

Many researchers have reported the correlation between the TPC and antioxidant capacity, particularly in case of using very popular chemical in vitro assays such as DPPH^•^/ABTS^•^^+^ (2,2′-azinobis-(3-ethylbenzothiazoline-6-sulfonate) scavenging, FRAP (ferric reducing antioxidant power) and LPO (inhibition of lipid peroxidation). The ethyl acetate (EtOAc) extract of *S. americana* berries and other nine edible North American plants were tested for antioxidant activity using the DPPH^•^ scavenging assay. DPPH^•^ scavenging value IC_50_ of *S. americana* was 113.96 µg/mL; other in this study investigated plants, *Gaultheria shallon* and *Sambucus cerulea* exhibited stronger antioxidant capacity with IC_50_ values of 14.76 and 29.32 µg/mL, respectively [36]. Methanol extracts of *S. americana* dried bark and leaves were remarkably stronger DPPH^•^ scavengers with EC_50_ of 15.80 μg/mL [12] and 38.76 μg/mL [53], respectively. For comparison, these values for black tea and coffee were 15.19 and 40.32 μg/mL, respectively [77].

Hukkanen et al. reported high antioxidant activity and phenolic contents in the fruits of several sweet rowanberry (*S. aucuparia*) cultivars, namely Burka, Dessertnaja, Eliit, Granatnaja, Kubovaja, Rosina, Rubinovaja, Titan, and Zholtaja: DPPH^•^ scavenging capacity and FRAP values were in the ranges of 9.7–21.3 g fw/g radical and 61–105 mmol Fe(II)/g fw [31]. Olszewska et al. analysed different anatomical parts of *S. aucuparia, S. aria* and *S. intermedia* and found that *S. aucuparia* inflorescence demonstrated the highest antioxidant capacity: in FRAP and ABTS^•+^ decolouration assays it was 2453.5 μmol TE/g dw and 83.05 mg/L, respectively, while the IC_50_ in DPPH^•^ scavenging assay was 18.05 μg/mL. Respective values of *S. aria* fruit were 497.7 μmol TE/g dw (FRAP), 142.20 mg/L (ABTS^•+^) and 95.31 μg/mL (DPPH^•^) [54].

The same authors measured DPPH^•^ scavenging of 16 *Sorbus* spp. and determined that the lowest EC_50_ values demonstrated methanolic extracts of *S. aucuparia, S. pohuashanensis, S. decora, S. koehneana, S. commixta, S. gracilis,* and *S. sitchensis* inflorescences and *S. wilfordinci, S. pogonopetala, and S. gracilis* leaves; they were in the ranges of 16.20–27.21 μg/mL and 15.23–20.71 μg/mL, respectively. These results correlated with high total phenolic levels [53]. Mrkonjić et al. [28] observed that methanolic and water extracts and jams of *S. aucuparia* fruits were stronger antioxidants than *S. torminalis.*

In order to identify the ability of different solvents to recover antioxidants from *S. aria* leaves various extracts were tested by DPPH^•^ scavenging method. Among the tested isolates, the EC_50_ value of ethyl acetate extract (2.99 mg/L), which contained 11.8% isoquercitrin, 6.0% astragalin, and 3.81% chlorogenic acid, was almost similar to reference antioxidant isoquercitrin, EC_50_ 2.76 mg/L [65]. Five strongly active constituents, namely isoquercitrin, rutin, quercetin 3-glucoside-7-rhamnoside, chlorogenic and neochlorogenic acids were found to be major components and principally responsible for the radical scavenging capacity of *S. aria* extracts [65]. Two interesting new coumarins, cashmins A (1) and B (2) were isolated from the methanolic extract of *S. cashmiriana*. Both compounds demonstrated outstanding antioxidant activity in H_2_O_2_ (IC_50_ 15.4 and 18.6 μmol/mL), as well as in ABTS^•+^ scavenging assays (IC_50_ 24.4 and 18.3 μmo/mL). For comparison, IC_50_ of ascorbic acid were 11.4 μmol/mL in H_2_O_2_ and 6.5 μmol/mL in ABTS^•+^ assays [15].

Bae et al. [69] tested the effects of treatment with carbohydrate hydrolases on the composition of TPC and flavonoids, as well as antioxidative activity of ethanol extract of dried *S. commixta* cortex. Amyloglucosidase, α-amylase, α-glucosidase and β-glucanase increased the contents of extractable polyphenols and flavonoids, as well as the DPPH^•^ scavenging capacity; particularly in case of applying β-glucanase [69].

Raudone et al. [71] detected twenty four constituents in the leaf samples of *S. anglica, S. aria, S. arranensis, S. aucuparia, S. austriaca, S. caucasica, S. commixta, S. discolor, S. gracilis, S. hostii, S. semi-incisa* and *S. tianschanica*, using ultra high performance liquid chromatography. Reducing activity of detected constituents was identified by the post-column FRAP assay; the highest total antioxidant activities of 175.3, 169.2 and 148.11 μmol TE/g dw were determined for *S. commixta, S. discolor* and *S. gracilis*, respectively.

Ethanol recovered antioxidants from *S. commixta* stems more effectively than hot water with the values of 504.39 and 364.64 μg/mg, respectively. Similarly, ethanol extracts demonstrated slightly higher antioxidant activities than water extracts in Fe^2+^ chelating, DPPH^•^, ABTS^•+^, hydroxyl and nitrite radical scavenging assays [68]. Extraction and fractionation of *S. domestica* fruits harvested at five different maturity stages gave the products with scavenging capacity in the range of 0.341–39.5 mg dwe/mg DPPH^•^ and the following order: water << dichloromethane < ethyl ether < ethyl acetate [58]. The fractions recovered with organic solvents possessed greater radical scavenging capacity than trolox, while the unripe fruits provided more antioxidants than the well-matured berries at room temperature. Finally, radical scavenging values strongly correlated with the total phenolic content in the fractions of *S. domestica* [58]. Olszewska et al. demonstrated that strong antioxidant fractions might be obtained from 70% methanol extracts of inflorescences and/or leaves of seven *Sorbus* spp. by using different solvents in a separatory funnel. *n*-Butanol and ethyl acetate gave the fractions with outstanding antioxidant capacity in the all applied assays: EC_50_ 3.2–5.2 μg/mL in DPPH^•^, 2.8–4.0 mmol TE/g in ABTS^•+^, 9.8–13.7 mmol Fe^2+^/g in FRAP and 39.6–58.2% GAE in Folin-Ciocalteu [37]. Consequently, properly selected solvents may provide promising antioxidants for food and medicinal applications.

The fruits and jam of *S. aucuparia* and two varieties of *S. torminalis*, were assayed for DPPH^•^, ^•^NO, HO^•^ and O_2_^•^ scavenging capacity, FRAP, and Fe^2+^/ascorbate induced LP inhibition. As already mentioned, *S. aucuparia* extracts were found to be the most effective almost in all tests, except for the assay toward the neutralisation of O_2_^•−^ when *S. torminalis* var. *torminalis* was the most potent. *S. torminalis* var. *torminalis* and *semitorminalis* showed similar antioxidant activity, however, var. *torminalis* had a slightly better antiradical power towards ^•^NO, O_2_^•−^ and HO^•^, while the extracts of *semitorminalis* acted more effectively in scavenging DPPH^•^, inhibiting LP and reducing Fe^2+^ [28]. Antioxidant capacity of extracts depends also on the nature of the assay as well as the polarity of solvent. For example, the results of Bobinaitė et al. demonstrated superior antioxidant capacity of *S. aucuparia* pomace water extract in the all test systems: in DPPH^•^, FRAP and ORAC (oxygen radical absorbance capacity) assays it was 309 μmol TE/g, 323 μmol TE/g and 263 mg TE/g, respectively; while ethanol extract was the next with its DPPH^•^ and ORAC values of 103 μmol TE/g and 201 mg TE/g, respectively [59].

## 6. Toxic Constituents of Rowanberries

Parasorbic acid, an important inhibitor of germination, has been reported in the fruits and seeds of *S. aucuparia* at the level of 4–7 mg/g and 0.08–0.12 mg/g fw, respectively [79]. This compound irritates the gastric mucosa and, if consumed at larger amounts, can cause indigestion and kidney damage to humans. However, heat treatment or freezing modifies the parasorbic acid into nontoxic sorbic acid. Parasorbic acid is sensitive to changes of temperature and brakes into safe compounds if the berries are picked after the first frost [34]. The parasorbic acid is almost absent in the cultivated hybrids. The other toxic component in rowanberries is the cyanogenic glycoside prunasin, which is derived from the amino acid phenylalanine; 1 g of the prunasin can liberate 91.5 mg HCN (hydrogen cyanide). Thus, HCN from the seeds of rowanberries, when formed at the levels exceeding 2–3 mg/L, can cause respiratory failure and even death [80]. Therefore, while processing the rowanberry pomace, the separation of the seeds would be essential.

## 7. Promising Health Benefits and Related Applications in Foods, Nutraceuticals and Pharmaceuticals

It is evident that among phytochemicals and other nutrients, polyphenolic compounds and ascorbic acid may be considered as the most valuable health beneficial constituents, which have been reported in various anatomical parts of *Sorbus* spp. The polyphenolics, which may influence the colour and flavour, have demonstrated antioxidant [54,81], antidiabetic [11,82] anti-hyperlipidemic [83], anti-inflammatory [84], antimicrobial [85], anticancer [86,87] antiviral [67], antifungal [79], antitumoral [88], anti-periodontal [89], and anti-osteoarthritis [90] effects, as well as vasoprotective [84], neuroprotective [26,91,92], cardioprotective [36], hepatoprotective [7], properties and COX-2 (cyclooxygenase-2) inhibitory [93] activities. Many of these activities are correlated to antioxidant capacity of bioactive compounds, which at cellular level may neutralize excessive reactive oxygen species, and thereby protect important biomolecules in the conditions of oxidative stress, which can cause cellular injury and development of chronic diseases. Therefore, it has been hypothesized that antioxidant-rich diets might play an important role in neutralising the excessive reactive oxygen species [11]. This hypothesis and increasing amount of evidence in favour of it have encouraged many researchers to test many novel plant-based phytochemicals as natural candidates for developing health beneficial exogenous antioxidants. The other important role of antioxidants is to protect foods and other sensitive to oxidation products during processing and storage in order to extend their shelf life and improve the quality and safety [94]. Compared to pure synthetic compounds natural preparations of phenolic antioxidants can be more effective due to the synergistic effects of various molecules present in the plant-based products. In addition, natural ingredients are usually safer than their synthetic counterparts and therefore are preferred by the consumers [11].

The application of plant-based polyphenolic substances in lipid-containing foods, cosmetics, and medicinal products is hampered by their high polarity (hydrophilicity), which makes them poorly soluble in the lipid medium, which is composed mainly of triacylglycerols. Therefore, for increasing product lipophilicity some studies [94,95,96] applied derivatisation of phenolic compounds by attaching medium or long chain alkyl molecules. For instance, the lipophilised phenolic extract of *S. aucuparia* was more effective inhibitor of rapeseed oil oxidation during 7-day storage than the untreated one: it reduced peroxide value by 43% and improved the solubility of the phenolics during frying [95]. Hydrophilic fraction of rowanberry pomace contained most of the polyphenolic antioxidants, while lipophilic seed extracts could be beneficial as nutraceutical and cosmetic agents due to the high content of polyunsaturated fatty acids and carotenoids [97].

Water extracts of berries containing high amounts of low molecular weight proanthocyanidins, which were tested as the inhibitors of colon cancer-induced angiogenesis, turned out to be superior in reducing Caco-2 cell viability [59]. Due to the significant content of bioactive phenolics in fruits, the wild rowanberries inhibited lipid oxidation both in liposomes and in emulsions [31]. Aqueous methanol extracts of *S. aucuparia* fruits were potent antioxidants while the extracts of both *S. torminalis* varieties, namely *torminalis* and *semitorminalis* effectively inhibited the growth of *E. coli*, var. *torminalis* being the best inhibitor of *Staphylococcus aureus* [28]. Polyphenols from two hybrid cultivars of *S. aucuparia*, Zoltaja and Granatnaja also delayed pathogenic *E. coli* growth. The phenolic extracts of wild rowanberries and cultivated breed Burka had an inhibitory effect on hemagglutination of *E. coli* HB101 (pRR7), which expresses the M hemagglutinin [60]. *S. aucuparia* berry extract isolated with acidified acetone also demonstrated high activity against *Salmonella enterica* ssp. *enterica* ATCC BAA-2162, and *Pseudomonas aeruginosa* ATCC 9027; it also exhibited moderate activity towards the two *Listeria monocytogenes* strains and *Proteus vulgaris* [66]. Water extract of *S. aucuparia* fruit inhibited the growth of Gram-positive *E. faecalis*, *S. aureus* and Gram-negative *S. enterica*, as well as the viability of *C. freundii* and *B. cereus* [59]. These findings prove that *S. aucuparia* extracts express strong antimicrobial activity against a wide scale of microorganisms and possess the high mitogenic activity expressed as the stimulating effect on hamster lymphocyte proliferation [66]. Water and methanol extracts of *S. aucuparia* fruits were also effective in the inhibition of acetylcholinesterase (AChE) and exhibited in vitro cytotoxicity in SRB assay, using tumour HeLa, MCF7 and HT-29 and healthy MRC-5 cell lines; however, they didn’t exhibit selectivity towards tumour cell lines [28].

The content of chlorogenic acid in sweet rowanberries can reach up to 200 mg/100 g, which is comparable with Arabica variety coffee beans, the richest source of this phenolic acid containing 280 mg/100 g [31]. Chlorogenic acids have been associated with a decreased risk of type 2 diabetes (T2D); they hydrolyse to caffeic acid, which reduces glucose absorption and oxidative stress in vitro and inhibits glucose-6-phosphate translocase, thereby decreasing glucose output in the liver. Intact chlorogenic acids are poorly absorbed in the small intestine, while the released after hydrolysis cinnamic acids are effectively absorbed with the help of enzymes in the colon depending on the precursor chlorogenic acid type and individual characteristics of a person [98]. Consequently, caffeoylquinic acid derivatives isolated from *S. commixta* fruits might be used for the regulation of diabetic complications and other pathogenic complications. These compounds also showed the most potent inhibitory effect against formation of the advanced glycation end products (AGE); neo-chlorogenic, crypto-chlorogenic, and chlorogenic acid exhibited potent inhibitory effects against peroxynitrite in radical scavenging assay [99].

Boath et al. [100] have reported that *S. aucuparia* fruits inhibited α-glucosidase with IC_50_ value 30 µg GAE/mL and were as effective as the pharmaceutical inhibitor acarbose for maintaining post-prandial glycemic control in T2D. Lately berry extracts of 16 different *Sorbus* species of subgenus *Sorbus* and *Aria* were tested for their α-amylase and α-glucosidase inhibitory activity. The study included *S.aucuparia, S. aucuparia* f. *xanthocarpa,, S.commixta, S. commixta* var. *rufo-ferruginea, S. decora, S. discolor, S. hybrida, S. koehneana, S. vilmorinii* and crossbreeds *S*. *aucuparia × americana, Sorbus × meinichii, Sorbus × splendida* (from subgenus *Sorbus*) and *S. alnifolia, S. folgneri, S. latifolia, S. minima, S. norvegica* (from subgenus *Aria*). The berry extract of *S. norvegica*, which belongs to subgenus *Aria*, also inhibited α-amylase and α-glucosidase and therefore was used in an oral starch tolerance test in streptozotocin (STZ)-treated C57BL/6 mice; administration of 900 mg extract daily demonstrated anti-hyperglycemic activity, which however was 36 times lower than in case of clinically used acarbose [24]. Thus, the berries of *S. norvegica* (subgenus *Aria)* may have some prospects in management T2D.

Twenty-nine different extracts, fractions and residues obtained from *S. domestica* fruits, harvested at 5 maturity phases were assessed for their in vitro inhibitory capacity of a rate-limiting enzyme aldose reductase [36,47]. Diethyl ether and ethyl acetate fractions effectively inhibited aldose reductase and the effect was associated with the high content of flavonoids and hydroxycinnamic acid esters determined in the extracts by liquid chromatography with diode array detector and mass spectrometer (LC-DAD-MS). The authors concluded that consumption of *S. domestica* fruit might be a promising way to reduce the occurrence of long-term complications of T2D, particularly at the early disease stages. The fractions of diethyl ether, ethyl acetate and dichloromethane of *S. domestica* were also noted as potential antioxidants to be used in food and medicinal preparations [36,47]. In addition, Bailie et al. [11] suggested that both flavonoids and terpenoids could offer benefits to treat a number of T2D symptoms.

Several studies reported the effects of *Sorbus* bioactives on cancer cell lines and some disease biomarkers. Thus, vanillic acid 4-*O*-α-L-rhamnopyranoside, protocatechuic acid anhydride and trivanilloyl-(1,3,4-trihydroxybenzoyl) ester, which were the predominant antioxidants of the *S. domestica* fruits, make it potentially useful for the mitigation of several diseases, such as *Clostridium difficile* infection, inflammatory bowel and irritable bowel syndrome [101]. Ethanolic *S. commixta* fruit extract remarkably reduced the viability of human lung cancer cell lines through the induction of apoptosis irrespective of their p53 status [102]. Another study reported that ethyl acetate fraction of *S. commixta* exhibited considerable inhibition against thrombin, prothrombin, blood coagulation factors and platelet aggregation, without haemolysis activity at the doses up to 0.5 mg/mL and therefore, has the potential to be used as a new anti-coagulation agent [103]. The juice of *S. sambucifolia* provoked differentiation of HL-60 leukemic cells to monocyte/macrophage characteristics in a concentration-dependent manner as denoted by histochemical and biochemical assays; it was suggested that these findings are promising for developing new agents suitable for differentiation therapy of leukaemia with fewer side effects [70]. The *S. umbellata* (Desf.) Fritsch var. *umbellata* leaf extract demonstrated dose-dependent cytotoxic effect to A549 and MCF-7 cells in MTT assay, while the highest cell proliferation inhibition was observed for the A549, 71.8% at 150 μg/mL [104].

Sakuranetin isolated from *S. commixta* plant actively inhibited rhinovirus-3 (HRV3) replication and at 100 μg/mL exhibited higher than 67% antiviral activity without cytotoxicity in epithelioid carcinoma cervix (HeLa) cells; hence, it may be promising in developing novel drugs for treating HRV3 infections [105]. Water extract of the dried *S. commixta* inner stem bark suppressed the production of ^•^NO and prostaglandins at the transcriptional levels, thus acting as an anti-inflammatory remedy for ear oedema formation, which was induced by arachidonic acid in mouse. A targeted blockage of protein kinase B translocation and its upstream signalling pathways was suggested as a possible therapeutic approach to develop anti-inflammatory drugs in the treatment of chronic diseases [84]. Furthermore, lupeol isolated, from the stem bark of *S. commixta* showed significant inhibitory effects on osteoclastogenesis; therefore, addition of *S. commixta* and lupeol could be used for bone diseases, such as osteoporosis, Paget’s disease, osteolysis associated with periodontal disease, and multiple myeloma [38].

The treatment of *S. commixta* cortexes by β-glucanase increased extract antioxidant activity, while its application resulted in the enhanced viability of human dermal fibroblasts exposed to ultraviolet (UV) light [69]. Furthermore, Kim et al. tested the leaf extract of *S. alnifolia*, among the others, to develop new natural cosmetic ingredients with antioxidant activity. As a result, the trials proved that the extract of *S. alnifolia* exhibited 87% inhibition of elastase activity when applied at 1 mg/mL. This result may provide the relevant application of plant-based inhibitors of general elastase in cosmetics with effects for UV-irradiated and dry skin [106].

The methanol extracts of the dried stems and twigs of *S. alnifolia* contributed for protection against chemically and genetically induced dopaminergic neurodegeneration. Moreover, methanol extract of *S. alnifolia* plant increased food-sensing functions in the dopaminergic neuron degraded worms by 58.4% hereby prolonging the average lifespan by 25.6%. Therefore, the extract of *S. alnifolia* can be a useful candidate for the treatment of Parkinson’s disease [26].

## 8. Conclusions and Further Perspectives

Polyphenolic antioxidants are among the most popular topics in characterisation of different *Sorbus* spp. anatomical parts. Since *Sorbus* polyphenols (proanthocyanidins, chlorogenic acid isomers and flavonols) are recognized as potent antioxidants and health beneficial phytochemicals, and considering the significant phenolic content in various *Sorbus* spp., it can be concluded that their products could be an excellent sources of natural antioxidants [54]. Such bioactives may be useful both for protecting foods against oxidation/microbial spoilage and providing health benefits to the consumers by incorporation of *Sorbus* preparations into functional foods, nutraceuticals and/or cosmeceuticals. The results of current review confirm the specific phenolic composition and antioxidant activity of different plant parts of numerous *Sorbus* spp. All parts of *S. commixta*, the fruit, leaves and inflorescences of *S. aria, S. aucuparia, S. sambucifolia,* the leaves and inflorescences of *S. gracilis* and *S. koehneana* and the leaves of *S. wilfordii* and *S. pogonopetala* may be distinguished as the materials demonstrating outstanding antioxidant effect.

However, more systematic studies are required for developing convenient and acceptable to consumer applications of *Sorbus* ingredients in foods and/or food supplements. Some studies have proved that the products of rowanberries demonstrate antioxidant activity and can be considered as a good source of antioxidants in the diet; however, these studies are not sufficient for wider applications of *Sorbus*. The production of functional foods and nutraceuticals from selected *Sorbus* spp. is envisaged to impart valuable biological effects, especially those related to immunity and health.

## Figures and Tables

**Table 1:** Botanical classification of selected *Sorbus* spp. and their uses.

Species and Varieties (Subgenus)	Food Uses of Fruits	Anatomical Part: Medicinal and Other Uses	Ref.
*S. alnifolia* (Sieb. & Zucc.) K. Koch. (*Aria*)		Twigs: treatment of neurological disorders as a traditional medicine in Korea	[26]
*S. americana* Marshall— American mountain ash (*Sorbus*; informal group *Commixtae*)		Bark: treatment diabetes hypo-glycaemic, vaso-relaxant, antitussive and tonic agent	[36]
*S. aria* L. Crantz—chess-apple (*Aria*)	Jellies, jams, brandy, liqueurs, conserves and vinegar, traditional bread flour extender	Fruit: diuretic, anti-inflammatory, anti-diarrhoeal, vasodilatory and vitamin agent; leaves: ethnomedical antidiarrheal ingredients; inflorescences and fruit: diuretic, laxative and emmenagogue; treatment of painful menstruation, constipation and kidney disorders	[11] [18] [37]
*S. aucuparia* L.—European mountain ash (*Sorbus*)	Alcohol beverages, jams, jellies, honey (floured dried fruit)	Traditional diuretic, anti-inflammatory, antidiarrheal (dried fruits), vasodilatory and an appetite- improving agent, source of vitamins, mild laxative	[18] [22] [7]
*S. cashmiriana* Hedl. (*Sorbus* series Multijugae)		Bark: tea made from its bark—to treat nausea, the bark preparation- to treat heart diseases; berries: to cure scurvy	[15]
*S. commixta* Hedl. (*Sorbus*; informal group *Commixtae*)		Stembark: for treating asthma, bronchitis, gastritis and oedema, anti-inflammatory, -atherosclerotic, -alcoholic, and vascular-relaxant effects, anti-atherogenic, for treating arthritis, hypoglycaemic, antitussive and tonic agent	[38] [14] [39] [13]
*S. decora* (Sarg.) C.K. Schneid—northern mountain ash (*Sorbus*; informal group *Commixtae*)		Leaves and bark- an antidiabetic medicine	[11]
*S. domestica* L. (*Cormus*)	Food ingredients	Traditional diuretic, anti-inflammatory, antidiarrheal (dried fruits), vasodilatory, antidiabetic and vitamin agents	[18] [40] [41]
*S. hybrida* L.—oakleaf mountain ash (*Aria* sect. *Aria × Sorbus*)		An ornamental tree in northern Europe	[42]
*S. pohuashanensis* (Hance) Hedl. (*Sorbus*)		Fruits, stems and bark: traditional Chinese medicine for the treatment of chronic tracheitis, tuberculosis and oedema	[16]
*S. sambucifolia* (Cham. & Schlecht.) M. Roem.—Siberian mountain ash (*Sorbus* Lucidae Kom.)	Alcohol beverages, jams, jellies, honey (floured dried fruit)	In avitaminosis, arteriosclerosis, as antipyretic or diuretic agent.	[43]
*S. scopulina* Greene—Greene’s mountain ash (*Sorbus*; informal group *Commixtae*)	Sometimes used in pies, preserves, or wine-making		[17]
*Sorbus* × *thuringiaca* (Ilse) Fritsch—mountain ash (*Aria* sect. *Aria* × *Sorbus*)		An ornamental tree	[44]
*S. tianschanica* Rupr. (*Sorbus* series Tianshanicae Kom.)		Leaves: asthma, ventricular myocytes, dyspnoea, tuberculosis and gastritis	[10]
*S. torminalis* (L.) Crantz var. *torminalis* (*Torminaria*)	Jams and ingredients for food and fodder	Traditional diuretic, anti-inflammatory, antidiarrheal (dried fruits), vasodilatory and vitamin agents	[18] [28] [40]
*S. torminalis* var. *semitorminalis* (*Torminaria*)		Traditional diuretic, anti-inflammatory, antidiarrheal (dried fruits), vasodilatory and vitamin agents	[18]

**Table 2:** Bioactive compounds in selected *Sorbus* species.

No.	Species, Tested Material and Its Isolation Method	Phenolic Acids: Total Amount (To) in GAE (%) or as Specified	Flavonoids/Proanthocyanidins in CyE (%) or as Specified	Ref.
1.	*S. americana*; 5 mL ME 10 mL ME 20 mL ME of F	All in mg/g dwe: To 3.599; 5-CQA 0.662; 3-CQA 2.837; QG 0.101 To 4.432; 5-CQA 0.714; 3-CQA 3.599; QG 0.119 To 5.388; 5-CQA 0.905; 3-CQA 0.417; QG 0.066		[55]
	*S. americana;* 70% ME of L	To 6.47; 5-CQA 0.04; 3-CQA 1.85	Fl: QU 0.46; KA 0.04; PAC 3.66	[53]
2.	*S. aria;* 70% ME of I, F & L	I: To 6.58; 5-CQA 1.18; 3-CQA 1.78 L: To 6.06; 5-CQA 0.99; 3-CQA 0.74 F: To 2.98; 5-CQA 0.32; 3-CQA 0.30	Fl in I: QU 0.277; SX 0.050; KA 0.041; IS 0.284 Fl in L: QU 0.493; SX 0.014; KA 0.242; IS 0.095 Fl in F: QU 0.009; KA 0.002; IS 0.007 PAC: I 2.75; L 3.53; F 1.80	[54]
	*S. aria;* ME of I, F & L		Fl, mg/100 g; F: Ag 20.3; Gl 31.1; QU 9.4; KA 2.4; IS 8.5 I: Ag 687.2; Gl 1049.0; QU 291.6; SX 52.6; KA 43.7; IS−299.3 L: Ag 888.1; Gl 1371.9; IS 99.8; QU 518.9; SX 14.8; KA 254.6	[18]
	*S. aria;* EtE of F	In mg/g dw; To: 3.91−10.81; 5-CQA: 0.18−4.00; 3-CQA: 0.22−2.30	Fl, µg/g dw; RU: 138.4–892.0; HY: 2.3–27.6; IQ: 10.9–108.6; QU: 2.1–35.2 PAC, mg/g dw: avr. 1.11	[57]
3.	*S. aucuparia;* 70% ME of I	To 21.17; DEF 37.61; EtAF 54.34; BF 48.71; WR 9.05		[43]
	*S. aucuparia;* AE of F	To 190 mg/100 g dw	Fl-To 68.1 mg/100 g dw	[49]
	*S. aucuparia;* 80% AE of F	To 0.2148; 5-CQA 0.0427; 3-CQA 0.0705	PAC 0.0005	[66]
	*S. aucuparia;* 70% AE of F	mg/g dw; wild F: 5-CQA 5.36; 3-CQA 8.59; other 1.84; HB 0.11. Cultivars: 5-CQA 2.23−7.31; 3-CQA 3.20−9.22; other 0.61−1.84; HB: 0.16−0.70	mg/g dw; wild: flavonols 1.84; flavanols 0.97; PAC 0.12. Cultivars: Fl 0.94–1.88; flavonols 0.95–1.89; PAC 0.36–6.04	[60]
	*S. aucuparia;* ME of F	Cultivars 4.35−8.19	Fl, g/kg fm: wild 3.11	[34]
	*S. aucuparia;* WE of F, ME of F & jam	µg/g dwe: WE-F: 3-CQA 5.69x10^3^; FA 7.8 ME-F: 3-CQA 5.80x10^3^; FA 9.59 Jam: PCA 12.5; 3-CQA 2.60x10^3^; FA 11.4	Fl, µg/ g dwe; WE-F: AF 10.7; KA-3-*O*-gl 9.0; QU-3-*O*-gl 49.3; HY 36.6; RU 82.3 ME-F: AF 11.9; KA-3-*O*-gl 8.56; QU-3-*O*-gl 55.8; HY 39.6; RU 80.4 Jam: AF 8.4; KA-3-*O*-gl 3.99; QU-3-*O*-gl 17.9; HY 9.68	[28]
	*S. aucuparia;* 70% AE of cultivars	In mg/ 100 g fw: To 550–1014; 5-CQA 34–104; 3-CQA 29–160	PAC 6–80 mg/100 g fw	[31]
	*S. aucuparia*; 70% ME of I, F & L	I: To 11.83; 5-CQA 1.37; 3-CQA 2.98 L: To 9.09; 5-CQA 1.15; 3-CQA 2.75 F: To 2.68; 5-CQA 0.29; 3-CQA 0.64	I: (Fl) QU 1.054; SX 0.151; KA 0.071. PAC 5.01 L: (Fl) QU 0.835; KA 0.188. PAC 3.84 F: (Fl) QU 0.051; KA 0.006. PAC 1.07	[54]
	*S. aucuparia* EtE of F	In mg/g dw: To 5.25–15.91; 5-CQ 0.67–7.03; 3-CQA 0.3510.01	Fl, µg/g dw: RU 40.1–598.3; HY 2.4–559.9; IQ 6.1–252.8; QU 2.8–83.5. PAC (avr.) 0.92 mg/g dw	[57]
	*S. aucuparia;* ME of I, F & L		Fl, mg/100 g: (L) Ag 1078; Gl 1666; QU 881.1; KA 196.9. (F) Ag 60.2; Gl 92.9; QU 53.8; KA 6.4. (I) Ag 1344.1; Gl 2067.4; QU 1110.7; SX 0.1582; KA 75.2	[18]
	*S. aucuparia;* 70% ME of I & L	I: To 10.02; 5-CQA 0.74; 3-CQA 2.27 L: To 8.23; 5-CQA 0.51; 3-CQA 1.90	Fl: (I) QU 1.048; SX 0.190; KA 0.084 (L) QU 0.903; KA 0.157. PAC: I 5.94; L 3.59	[53]
4.	*S. cashmiriana;* 70% ME of L	L: To 5.78; 5-CQA 0.37; 3-CQA 1.25	Fl: QU 0.532; KA 0.113. PAC 4.02	[53]
5.	*S. tianschanica;* 50% EtE of F& L	F, mg/g: 5-CQA 3.7; 3-CQA 2.6. L.: 5-CQA 6.0; 3-CQA 7.0	Fl, mg/g: (F) RU 0.15; HY 0.08; IQ 0.32. (L) RU 1.5; HY 1.4; IQ 5.1	[56]
	*S. tianschanica;* WE of L		Fl, mg/g: RU 0.71; HY 1.18; HE 0.48	[67]
6.	*S. commixta;* 50% EtE of L & F	L, mg/g: To 35.74; 5-CQ-.1.10; 3-CQA-21.91. F: To-11.19; 5-CQA- 1.8; 3-CQA-7.5	Fl, mg/g: L: HY-7.5; IQ-5.3. F: HY-1.20; IQ-0.65; RU-0.02	[56]
	*S. commixta;* hot-WE and 70% EtE of S	To in µg/mg: We 364.64; EtE 504.39	To-Fl, µg/mg: WE 124.59; EtE 160.09	[68]
	*S. commixta;* 70% EtE of C	To, µg/mg: Without enzyme 447.3; treated with: amylase 501.6; amyloglucosidase 461.2; glucosidase 510.7; glucanase 493.3; cellulase 449.6	Fl, µg/mg: without enzyme 35.1; treated with: amylase 55.1; amyloglucosidase 41.4; glucosidase 51.3; glucanase 63.0; cellulase 36.8	[69]
	*S. commixta;* 70% ME of I and fractions (f)	ME 21.17; DEf 37.61; EtAf 54.34; Buf 48.71; WR 9.05		[37]
	*S. commixta;* 70% ME of I & L	I: To 9.29; 5-CQ 0.76; 3-CQA 3.92 L: To 8.08; 5-CQ 0.05; 3-CQA 0.79	Fl: (I) QU 0.422; KA 0.050; SX 0.045 (L) QU 0.470; KA 0.011. PAC: I 5.98; L 3.58	[53]
7.	*S. decora;* 70% ME of I & L	I: To 11.67; 5-CQA 1.26; 3-CQA 3.85 L: To 8.10; 5-CQA 0.19; 3-CQA 2.10	Fl: (I) QU 0.839; KA 0.059; SX 0.07. (L) QU 0.474; KA 0.035. PAC: I 6.40; L 4.03	[53]
	*S. decora;* 70% ME of I	ME 24.61; DEf 34.50; EtAf 55.16; Buf 53.75; WR 10.06		[37]
8.	*S. domestica;* ME of (1), (2), (3), (4), (5)	To, µg/mg: R: 13.6 (1) → 25.4 (2) → 20.5 (3) → 32.1 (4) → 30.2 (5); DCMF: 74.5 (1) → 27.0 (2) → 97.0 (3) → 66.5 (4); DEf: 245(1) →151(2) → 324 (3) → 148(4) → 143 (5); EtAf: 285 (1) → 137 (2) → 198 (3) → 64(4) → 341(5); Buf: 94.0 (1) → 16.1 (2) → 25.1 (3) → 12.5 (4) →140 (5); Wf: 14.8 (1) → 3.03 (2) → 11.3 (3) → 2.27 (4) → 34.4 (5); ME: 32.5 (1) → 10.3 (2) → 26.3 (3) → 5.58 (4) → 28.1 (5)		[58]
	*S. domestica;* ME of (1), (2), (3), (4), (5)	(1): To 14.72; CiA 10.55; BA 4.17. (2): To 18.85; CiA 9.91; BA 8.94. (3): To 18.18; CiA 14.24; BA 4.57. (4): To 19.28; CiA 12.19; BA 7.09. (5): To 4.86; CiA 2.55; BA 2.31	Fl: (1) To 8.68; Ag 1.22; Gl 7.46. (2) To 3.08; Ag 0.36; Gl 2.72. (3) To 10.59; Ag 1.46; Gl 8.83. (4): To 2.45; Ag 0.46; Gl 1.99. (5) To 7.9; Ag 0.73; Gl 7.17	[41]
9.	*S. gracilis;* 70% ME of I & L	I: To 11.06; 5-CQA 0.19; 3-CQA 3.31 L: To 10.72; 5-CQA 0.03; 3-CQA 0.93	Fl: (I) QU 0.194; KA 0.012; SX 0.072 (L) QU 0.113; KA 0.008. PAC: I 6.54; L 6.56	[53]
	*S. gracilis;* 70% ME of I & L	I: ME 24.63; DEf-36.87; EtAf 54.09; Buf 57.09; WR 8.21. L: ME 30.62; DEf 34.90; EtAf 52.37; Buf 48.62; WR 11.45		[37]
10.	*S. intermedia;* ME of I, F & L		Fl, mg/100g: (I) Ag 1514.8; Gl 2320.7; QU 1053.4; SX 117.3; KA 29.3; IS 314.8. (L) Ag 424.1; Gl 652.6; QU 303.6; KA 52.0; IS 68.5 (F) Ag 44.4; Gl 68.2; QU 32.5; IS 9.5; KA 2.4	[18]
	*S. intermedia* 70% ME of I, F & L	I: To 9.25; 5-CQA 0.68; 3-CQA 2.35 L: To 8.74; 5-CQA 0.65; 3-CQA 1.26 F: To 2.24; 5-CQA 0.27; 3-CQA 0.23	Fl: (I) QU 0.277; SX 0.05; KA 0.041; IS 0.284. PAC 5.52 (L) QU 0.493; SX 0.014; KA 0.242; IS 0.095. PAC 5.45 (F) QU 0.009, KA 0.002; IS 0.007. PAC 0.82	[54]
11.	*S. koehneana;* ME of I & L	I: To 11.67; 5-CQA 1.98; 3-CQA 2.05 L: To 9.87; 5 CQA-0.53; 3-CQA 1.97	Fl: (I) QU 0.27; KA 0.02; SX 0.05. PAC 6.86 L: QU 0.25; KA 0.11. PAC 5.81	[53]
	*S. koehneana;* 70% ME of I & L	To: ME 26.38; DEf32.10; EtAf50.51; Buf 58.17; WR 10.51		[37]
12.	*S. pohuashanensis;* 70% ME of I & L	I: To 11.32; 5-CQA 0.7; 3-CQA 2.48 L: To 6.26; 5-CQA 0.12; 3-CQA 0.67	Fl: I: QU-0.4; KA-0.04; SX-0.02. L: QU-0.12; KA-0.03. PAC: I-7.67; L-3.93	[53]
13.	*S. pogonopetala;* 70% ME of L	To 10.9; 5-CQA 0.22; 3-CQA 1.63	Fl: QU 0.38; KA 0.26. PAC 5.89	[53]
	*S. pogonopetala;* 70% ME of L	To: ME 24.03; Def 42.85; EtAf 53.29. Buf 39.56; WR 10.38		[37]
14.	*S. sambucifolia;* 70% ME of I & L	I: To 8.2; 5-CQA 0.42; 3-CQA 4.17. L: To 5.07; 5-CQA 0.1; 3-CQA 1.02	Fl: (I) QU 0.81; KA 0.06; SX 0.13. PAC 3.79 (L) QU 0.16; KA 0.01. PAC 1.96	[53]
	*S. sambucifolia;* EtE of F	To 0.733	Fl: To 0.002	[70]
15.	*S. scalaris;* 70% ME of I & L	I: To 8.47; 5-CQA 0.6; 3-CQA 2.36 L: To 4.23; 5-CQA 0.36; 3-CQA 1.24	Fl: (I) QU 0.34; KA 0.06; SX 0.15. PAC 5.68 (L) QU 0.22; KA 0.13. PAC 1.47	[53]
16.	*S. setschwanensis;* 70% ME of L	To 10.18; 5-CQA 0.22; 3-CQA 2.61	Fl: QU 0.57; KA 0.31. PAC 5.56	[53]
17.	*S. sitchensis;* 70% ME of I & L	I: To 10.08; 5-CQA 0.45; 3-CQA 3.13 L: To-4.89; 5-CQA- 0.05; 3-CQA-0.56	Fl: (I) QU 0.38; KA 0.02; SX 0.05 L: QU-0.27; KA-0.02. PAC: I-7.14; L-1.48	[53]
18.	*S. torminalis* var. *torminalis;* WE of F, ME of F & jam	In µg/g dwe; WE-F: PCA 13.7; FA 27.8 ME-F: PCA 23.2; FA 62.6 Jam: PCA 5.92; FA 13.3	Fl, µg/g dwe; WE-F: AF 15.8 ME-F: AF 19.3; QU-3-*O*-gl 13.6; HY 10.4 Jam: AF 16.8; QU-3-*O*-gl 2.53; HY 1.61	[28]
	*S. torminalis* var. *semitorminalis;* WE of F, ME of F & jam	WE-F: GA 5.69; FA 43.3; PCA 4.61 ME-F: FA 38.3; PCA 3.44 Jam: FA 18.4; PCA 2.11	Fl, µg/g dwe; WE-F: AF 362; KA-3-*O*-gl 2.34; QU 6.53; QU-3-*O*-gl 3.33; Cat 10.6 ME-F: AF 974; KA-3-*O*-gl 2.43; QU 11; QU-3-*O*-gl 2.06 Jam: AF 195; QU 3.76; QU-3-*O*-gl 1.60	[28]
19.	*S. wilfordii;* 70% ME of L	To 12.31; 5-CQA 0.13; 3-CQA 2.58	Fl: QU-0.88; KA-0.05. PAC: 5.31	[53]
	*S. wilfordii;* 70% ME of L	ME 29.93; DEf 53.13; EtAf 54.34; Buf 48.37; WR 15.27		[37]

F—fruits; L—leaves; I—inflorescences; S—stems; C—cortex, B—bark. M—methanol; Et—ethanol; A—acetone; DCM—dichloromethane; DE—diethyl ether; Bu—butanol; EtA—ethyl acetate; W—water; E—extract; R—residue; f—fraction. Total phenolic content is expressed in GAE (gallic acid equivalents); avr—average; fm—fresh mass; Fl—flavonoids in %; PAC—proanthocyanidins in % of CyE (cyanidin chloride equivalents); 3-CQA—chlorogenic acid; 5-CQA—neochlorogenic acid; GA—gallic acid; HC—hydroxycinnamic acid; CA—caffeic acid; p-c—p-coumaric; HB—hydroxybenzoic; Gl—glycoside, Ag—aglycone, PCA—protocatechuic acid; CiA—cinnamic acids, BA—benzoic acids; FA—ferulic acid; AF—amentoflavone; QG—quercetin-3-*O*-glucoside; KA-3-*O*-gl—kaempferol-3-*O*-glucoside; Cat—catechin; QU-3-*O*-so—quercetin-3-*O*-β-sophoroside; QU—quercetin; KA—kaempferol; SX—sexangularetin. Unripe fruit (1), well matured on tree (2), matured for 1 week at room temperature (3), matured for 3 weeks at room temperature (4), fruit pulp from well matured fruits (5).

**Table 3:** Bioactivity of selected *Sorbus* species.

No.	Species, Tested Material and Its Isolation Method	Antioxidant Activity EC_50_ (µg/mL) or as Specified	TEAA, mmol/g or LPO%	FRAP, mmol Fe^2+^/g or as Specified	Ref.
1.	*S. alnifolia*; 75% EtE of L	DPPH^•^ 30.6			[39]
2.	*S. americana*; 70% ME of L	DPPH^•^ 38.76	TEAA-0.34; LPO-54.29		[53]
	*S. americana*; EtAE of F	DPPH^•^ 113.9			[12]
	*S. americana* ME of B	DPPH^•^ 15.8			[36]
3.	*S. aria*; 70% ME of I, L & F	DPPH^•^: I 42.05; L 50.17; F 95.31	TEAA: I 0.41; L 0.344; F 0.18	I 1.394; L 1.119; F 0.498	[54]
	*S. aria* EtE of F	DPPH^•^, mg/mL: 0.49−2.50			[57]
4.	*S. aucuparia*; 70% ME	DPPH^•^: ME 8.93; DEf 5.53; EtAf 3.37; Buf 3.52; WR 9.96	TEAA: ME 1.72; DEf 2.14; EtAf 3.22; Buf 3.58; WR 0.94	ME 4.43; DEf 9.30; EtAf 12.77; Buf 10.84; WR 2.58	[37]
	*S. aucuparia*; ME of F and cultivars	ME-F, DPPH^•^, g/kg fm: 6.73; % of inhibition: HO^•^ 16.33; O_2_^•^ 26.74; ^•^NO 24.75. Cultivars: DPPH^•^ 6.58−9.62; % of inhib.: HO^•^ 16.12–24.73; O_2_^•^ 27.19–34.02; ^•^NO 25.03–31.39	ME-F LPO, % of inhibition: 8.21 Cultivars: 7.93–13.12		[34]
	*S. aucuparia*; AE of F	DPPH^•^, mmol/kg dw: 357		mmol Fe^2+^/ kg dw: 315.5	[49]
	*S. aucuparia*; WE of F, ME of F & jam	WE: DPPH^•^ 70; ^•^NO 1430; O_2_^•^ 20.16 × 10^6^; HO^•^ 160 ME: DPPH^•^ 80; ^•^NO 430; O_2_^•^ 20.5 × 10^6^; HO^•^ 240 Jam: DPPH^•^ 130; ^•^NO 2260; O_2_^•^ 67.8 × 10^6^; HO^•^ 610	LPO mg mL: WE-F - 6.40; ME-F - 7.38; jam - 4.08	mg of AAE/g: WE-F: 10.6. ME-F: 11.2. Jam: 4.22	[28]
	*S. aucuparia* (sweet cultivars); 70% AE	DPPH^•^, g/g: 21.3−9.7		0.061−0.105	[31]
	*S. aucuparia*; 70% ME of I, L, & F	DPPH^•^: I 18.05; L 27.47; F 163.63	TEAA: I 0.956; L 0.628; F 0.106	I 2.454; L 2.148; F 0.442	[54]
	*S. aucuparia*; 70% ME of I & L	DPPH^•^: I 16.69; L 24.10	TEAA: I 0.78; L 0.54 LPO: I 68.34; L 58.69		[53]
	*S. aucuparia* EtE of F	DPPH^•^ 340−4260			[57]
5.	*S. cashmiriana*	In µmol/mL; DPPH^•^ 7.6−12.5; H_2_O_2_ 15.4−18.6; ABTS^•+^ 18.3−24.4		µmol/mL:11.3−23.8	[15]
	*S. cashmiriana; 70*% ME of L	DPPH^•^ 48.59	TEAA 0.27; LPO 53.59		[53]
6.	*S. commixta*; hot-WE of S	In % of inhibition: (50 µg/L) ^•^OH 10.37; ^•^NO 92.63 (pH1.2), 66.82 (pH3). (25 µg/L) ^•^OH 10.08; ^•^NO 65.36 (pH1.2); 41.06 (pH3). (12.5 µg/L) ^•^OH 7.63; ^•^NO 42.59(pH1.2), 26.78 (pH3). (10 µg/L): DPPH^•^ 21.39; ABTS^•+^ 43.21. (5 µg/L) DPPH^•^ 12.75; ABTS^•+^ 24.96. (1 µg/L) DPPH^•^ 5.27; ABTS^•+^ 8.77		In %: 50 µg/L 19.28; 25 µg/L 9.28 12.5 µg/L 6.83	[68]
	*S. commixta*; 70% EtE of S	In % of inhibition: (50 µg/L) ^•^OH 23.61; ^•^NO 96.64 (pH1.2), 82.51 (pH3). (25 µg/L) ^•^OH 22.15; ^•^NO 91.97 (pH1.2), 80.02 (pH3). (12.5 µg/L) ^•^OH 18.42; ^•^NO 86.55 (pH1.2), 72.44 (pH3). (10 µg/L) DPPH^•^ 26.36; ABTS^•+^ 59.64. (5 µg/L) DPPH^•^ 15.96; ABTS^•+^ 37.01. (1 µg/L) DPPH^•^ 6.93; ABTS^•+^ 12.14.		In %: 50 µg/L 13.06 25 µg/L 10.31; 12.5 µg/L 9.30	[68]
	*S. commixta*; 70% EtE of C	Without enzyme: O_2_^•^ 14.2; DPPH^•^ 18.0. Amylase: O_2_^•^ 14.8; DPPH^•^ 15.4. Amyloglucosidase: O_2_^•^ 14.2, DPPH^•^ 15.8. Glucosidase: O_2_^•^ 13.8, DPPH^•^ 15.7. Glucanase: O_2_^•^ 13.6, DPPH^•^ 15.2. Cellulase: O_2_^•^ 14.6, DPPH^•^ 18.2			[69]
	*S. commixta*; 70% ME, f and R	DPPH^•^: ME 7.16; DEf 5.72; EtAf 3.52; Buf 3.53; WR 9.66	TEAA: ME 1.70; DEf 2.14; EtAf 2.62; Buf-3.40; WR 1.26	ME 5.04; DEf 7.58; EtAf 12.23; Buf 11.01; WR 2.70	[37]
	*S. commixta*; 70% ME of I & L	DPPH^•^: I 23.22; L 28.56	TEAA: I 0.56, L 0.46 LPO: I 78.21, L 58.65		[53]
7.	*S. decora*; 70% ME, Fs and R of I	DPPH^•^: ME 7.76; DEf 5.57; EtAf 3.44; Buf 3.17; WR 9.84	TEAA: ME 1.79; DEf 2.67; EtAf 3.98; Buf 3.55; WR 1.21	ME 5.42; DEf 8.5; EtAf 13.74; Buf 11.47; WR 2.77	[37]
	*S. decora*; 70% ME of I & L	DPPH^•^: I 16.20; L 27.21	TEAA: I 0.81; L 0.48 LPO: I 70.99; L 59.99		[53]
8.	*S. domestica*; ME of (1), (2), (3), (4), (5)	DPPH^•^: (R) 4829(1)→6290(2)→3720(3)→ 2730(4)→1810(5). DCMf: 3600(1)→ 9880(2)→3820(3)→6010(4). DEf: 997(1) →1740(2)→825(3)→3280(4)→ 2970(5). (EtAf) 1780(1)→1750(2)→1840(3)→3170 (4)→899(5). (Buf) 588(1)→8000(2)→3750 (3)→13200(4)→341(5). Wf: 4950(1)→ 39100(2)→5570(3)→39500(4)→2170(5). (ME) 2550(1)→10600(2)→1890(3)→ 20000(4)→1450(5)			[58]
9.	*S. gracilis*; 70% ME of I & L	DPPH^•^: (I) ME 7.93; DEf 5.39; EtAf 3.71; Buf 3.25; WR 10.12. (L) ME 6.60; DEf 5.29; EtAf 3.70; Buf 3.83; WR 9.54	TEAA: (I) ME 1.99; DEf 2.71; EtAf 3.65; Buf 3.68; WR 1.15. (L) ME 2.12; DEf 2.14; EtAf 3.72; Buf 3.33; WR 1.31	I: ME 5.36; DEf 9.34; EtAf 13.06; Buf-9.92; WR 2.26. (L) ME 6.2; DEf 8.72; EtAf 12.94; Buf 11.05; WR 2.98	[37]
	*S. gracilis*; 70% ME of I & L	DPPH^•^: I 19.09; L 20.71	TEAA: I 0.68; L 0.63 LPO: I 73.01; L 70.72		[53]
10.	*S. intermedia*; 70% ME of I, L & F	DPPH^•^: I 25.41; L 30.71; F 198.69	TEAA: I 0.679; L 0.572; F 0.087	I 2.123; L 1.512; F 0.298	[54]
11.	*S. koehneana*; 70% ME of I & L	DPPH^•^: I 16.20; L 24.74	TEAA: I 0.81; L 0.53 LPO: I 73.34; L 54.15		[53]
	*S. koehneana*; 70% ME of I	DPPH^•^: ME 6.74; DEf 5.70; EtAf 3.46; Buf 3.15; WR 9.71	TEAA: ME 2.08; DEf 2.60; EtAf 3.56; Buf 3.94; WR 1.29	ME 5.44; DEf 8.38; EtAf 12.87; Buf 9.81; WR 2.54	[37]
12.	*S. pohuashanensis*; 70% ME of I & L	DPPH^•^: I 17.89; L 43.86	TEAA: I 0.73; L 0.30 LPO: I 68.69; L 50.21		[53]
13.	*S. pogonopetala*; 70% ME of L	DPPH^•^: ME 6.84; DEf 4.89; EtAf 3.8; Buf 5.18; WR 9.83	TEAA: ME 1.81; DEf 2.28; EtAf 3.44; Buf 2.96; WR 1.03	ME 5.54; DEf 10.92; EtAf 11.42; Buf 8.67; WR 2.92	[37]
	*S. pogonopetala*; 70% ME of L	DPPH^•^ 19.87	TEAA 0.66; LPO 74.73		[53]
14.	*S. sambucifolia*; 70% ME of I & L	DPPH^•^: I 28.03; L 52.63	TEAA: I 0.47; L 0.25 LPO: I 58.12; L 54.03		[53]
15.	*S. scalaris*; 70% ME of I & L	DPPH^•^: I 27.65; L 57.86	TEAA: I 0.47; L 0.23 LPO: I 55.23; L 41.70		[53]
16.	*S. setschwanensis*; 70% ME L	DPPH^•^ 23.30	TEAA 0.56; LPO 63.77		[53]
17.	*S. sitchensis*; 70% ME of I & L	DPPH^•^: I 20.75; L 54.23	TEAA: I 0.63; L 0.24 LPO: I 68.26; L 53.13		[53]
18.	*S. torminalis* (L.) Crantz var. *torminalis*; WE of F, ME of F & jam	WE-F: DPPH^•^ 1380; O_2_^•^ 7.09×10^6^; HO^•^ 300. ME-F: DPPH^•^ 570; O_2_^•^ 12.2 × 10^6^, ^•^NO 2820; HO^•^ 260. Jam: DPPH^•^ 440; ^•^NO 640; O_2_^•^ 36.9 × 10^6^; HO^•^ 1110		mg AAE/g: WE-F 1.11; ME-F 2.12; jam: 3.1	[28]
19.	*S. torminalis* var. *semitorminalis*; WE of F, ME of F & jam	WE-F: DPPH^•^ 1270; O_2_^•^ 12.8 × 10^6^; HO^•^ 430. ME-F: DPPH^•^ 420; ^•^NO 3.12; O_2_^•^ 12.5 × 10^6^; HO^•^ 270. Jam: DPPH^•^ 180; ^•^NO 2.45; O_2_^•^ 50.3 × 10^6^; HO^•^ 290	LPO, mg mL: jam 3.02	mg AAE/g: WE-F 2.12; ME-F 3.81; jam 6.41	[28]
20.	*S. wilfordii*; *70*% ME of L	DPPH^•^: ME 6.01; DEf 3.67; EtAf 3.45; Buf 3.28; WR 9.04	TEAA: ME 2.24; DEf 2.97; EtAf 3.41; Buf 2.83; WR 1.51	ME 6.78; DEf 11.60; EtAf 12.55; Buf 10.99; WR 4.03	[37]
	*S. wilfordii*; 70% ME of L	DPPH^•^ 15.23	TEAA: L-0.86. LPO-86.92		[53]

DPPH^•^—2,2-diphenyl-1-picrylhydrazyl free radical scavenging capacity; ABTS^•^^+^—2,2′-azinobis-(3-ethylbenzothiazoline-6-sulfonate) radical cation decolouration assay; TEAA—trolox equivalent antioxidant activity, mmol/g; LPO—inhibition of lipid peroxidation, %; FRAP—ferric reducing antioxidant power. E—extract; f—fraction; R—residue; Et—ethanol; M—methanol; DE—diethyl ether; Bu—butanol; W—water; EtA—ethyl acetate; A—acetone; DCM—dichloromethane. I—inflorescences; L—leaves; F—fruits; S—stems; C—cortex, B—bark; fm—fresh mass. Unripe fruit (1), well matured on tree (2), matured for 1 week at room temperature (3), matured for 3 weeks at room temperature (4), fruit pulp from well matured fruits (5).
